# The association of grip strength from midlife onwards with all-cause and cause-specific mortality over 17 years of follow-up in the Tromsø Study

**DOI:** 10.1136/jech-2015-206776

**Published:** 2016-05-26

**Authors:** Bjørn Heine Strand, Rachel Cooper, Astrid Bergland, Lone Jørgensen, Henrik Schirmer, Vegard Skirbekk, Nina Emaus

**Affiliations:** 1 Norwegian Institute of Public Health, Oslo, Norway; 2 Institute of Health and Society, University of Oslo, Oslo, Norway; 3 Norwegian National Advisory Unit on Ageing and Health, Vestfold Hospital Trust, Tønsberg, Norway; 4 Department of Geriatric Medicine, Oslo University Hospital, Oslo, Norway; 5 MRC Unit for Lifelong Health and Ageing, University College London (UCL), London, UK; 6 Oslo and Akershus University College, Oslo, Norway; 7 Department of Health and Care Sciences, UiT The Arctic University of Norway, Tromsø, Norway; 8 Department of Clinical Therapeutic Services, University Hospital of North Norway, Tromsø, Norway; 9 Department of Clinical Medicine, Faculty of Health sciences, The Arctic University of Norway, Tromsø, Norway; 10 Division of Cardiothoracic and Respiratory Medicine, University Hospital of North Norway, Tromsø, Norway

**Keywords:** Epidemiology of ageing, LONGITUDINAL STUDIES, MORTALITY, PHYSICAL FUNCTION

## Abstract

**Background:**

Grip strength has consistently been found to predict all-cause mortality rates. However, few studies have examined cause-specific mortality or tested age differences in these associations.

**Methods:**

In 1994, grip strength was measured in the population-based Tromsø Study, covering the ages 50–80 years (N=6850). Grip strength was categorised into fifths, and as z-scores. In this cohort study, models with all-cause mortality and deaths from specific causes as the outcome were performed, stratified by sex and age using Cox regression, adjusting for lifestyle-related and health-related factors.

**Results:**

During 17 years of follow-up, 2338 participants died. A 1 SD reduction in grip strength was associated with HR=1.17 (95% CI 1.12 to 1.22) for all-cause mortality in a model adjusted for age, gender and body size. This association was similar across all age groups, in men and women, and robust to adjustment for a range of lifestyle-related and health-related factors. Results for deaths due to cardiovascular disease (CVD), respiratory diseases and external causes resembled those for all-cause mortality, while for cancer, the association was much weaker and not significant after adjustment for lifestyle-related and health-related factors.

**Conclusions:**

Weaker grip strength was associated with increased all-cause mortality rates, with similar effects on deaths due to CVD, respiratory disease and external causes, while a much weaker association was observed for cancer-related deaths. These associations were similar in both genders and across age groups, which supports the hypothesis that grip strength might be a biomarker of ageing over the lifespan.

## Introduction

Grip strength is a simple measure used to indicate overall muscle strength which has been shown to have health-related prognostic value.^1^ ^2^ Evidence suggests grip strength declines from midlife onwards^3–6^ and is a powerful predictor of future disability, falls, morbidity and mortality.^1^ ^7–9^ A systematic review and meta-analysis published in 2010 identified 23 publications which had examined the association between grip strength and all-cause mortality in community-dwelling populations.^9^ Effect estimates from 14 of these studies representing more than 50 000 participants were included in a meta-analysis, producing an overall summary HR of 1.7 for all-cause mortality when the weakest quarter of grip strength was compared with the strongest quarter.^9^ When meta-analyses were stratified by age, gender and length of follow-up, there was some evidence to suggest that associations between grip strength and all-cause mortality may be higher in older age groups, stronger in males compared with females, and in studies with follow-up of <10 years, however, there was limited statistical power to formally test these differences.^9^ Since the publication of this review, subsequent studies have continued to find that weaker grip strength is associated with increased all-cause mortality rates in a range of settings.^10–13^

Most studies of grip strength in middle and old age have investigated associations with all-cause mortality, and studies that have examined cause-specific mortality are not as numerous.^14–19^ Furthermore, often such studies are only powered to investigate broad groups of causes of death such as cardiovascular disease (CVD) and cancer, and have not been able to look at additional causes such as ischaemic heart disease (IHD), respiratory diseases and external causes. Recently, findings from a study which had measures of grip strength in almost 140 000 people aged 35–70 years from 17 countries were reported. This study found that weak grip strength was related to increased rates of all-cause mortality, cardiovascular mortality, myocardial infarction and stroke, while no association was found for cancer mortality.^14^ These findings of an association between grip strength and cardiovascular mortality and no association with cancer mortality are consistent with those from a study of 1 million Swedish male adolescents aged 16–19 years followed over a period of 24 years.^19^ These results, of an association between grip strength and cardiovascular mortality and no association for cancer, suggest a potential mechanistic explanation related to the cardiovascular system,^7^ ^14^ which should be further explored.

Large-scale studies including objectively measured physical performance and strength are lacking in Norway, with one exception; the Tromsø Study, a longitudinal study with six follow-ups in the period 1974–2008. Grip strength was objectively measured in 1994, in a sample of men and women covering a wide range of ages.^20^ Our aim was to investigate the associations of grip strength with all-cause and cause-specific mortality (CVD, stroke, IHD, cancer, respiratory diseases and external causes) in men and women in this large general Norwegian population. Low grip strength in adolescents has been found to be associated with increased suicide risk in young adulthood,^19^ but to the best of our knowledge, our paper is the first to investigate the association between grip strength and the broader group of external causes of death. Another novel aspect of our study was our aim to formally test whether any associations varied by age and sex at strength assessment.

## Methods

### Study population

The Tromsø Study is a longitudinal population-based multipurpose study focusing on lifestyle-related diseases, initiated in 1974 with surveys, inviting earlier participants as well as recruiting new participants, repeated in 1979, 1986, 1994, 2001 and 2008.^20^ This study uses baseline data from the survey initiated in 1994. At this time, all those men and women in the municipality aged 25 years and above (n=37 558) were invited to participate in phase I of the survey. All men aged 55–74 years, all women aged 50–74 years and 5–10% random samples of other birth cohorts aged 25–84 years were preselected to undergo a more extensive phase II examination (10 542 participants) which included grip strength measurements. The attendance rate was 74% in men and 77% in women. In the current analysis, 6850 participants (58% women) aged 50–80 years at the time of screening and with valid grip strength were included. Participants outside this age range were excluded as there were too few deaths among the 925 individuals below 50 years (n=41), and there were few participants above 80 years (n=14). The Regional Committee of Research Ethics recommended and the Norwegian Data Inspectorate approved the study.

### Assessment of grip strength

Grip strength of the non-dominant hand was measured in bar in the Tromsø Study, wave four (1994–1995) using a Martin vigorimeter. Each participant was allowed two attempts, and the highest score registered was recorded and used in analyses.

### Ascertainment of mortality

Data on each participant were linked to death certificate data from the Norwegian Cause of Death Registry, and participants were followed from 1 January 1996 to 31 December 2012, death or emigration, whichever occurred first. Thus, follow-up started ∼2 years after the time point of the health examination, which was done to minimise the effect of reverse causation from disease at baseline. Only 51 participants (0.7%) left the study (died or emigrated) during the first 2 years, before 1 January 1996. Participants had mean follow-up time of 14.4 years, and maximum 17.0 years, and mean age at the end of follow-up was 77.5 years (minimum 51.7 and maximum 96.4 years). Causes of death were based on underlying cause of death at the death recorded by a medical doctor on the certificate and categorised using the International Classification of Diseases (ICD) V.10 and grouped as: cancer (C00-C97), CVD (I00-I99), IHD (I20-I25), stroke (I60-I69), respiratory causes (J00-J99) and external causes (V01-Y89) (such as accidents, falls, injuries, poisoning, assault and other adverse events).

### Covariates

Covariates known to be associated with grip strength and mortality were selected a priori for inclusion as possible confounders. All these covariates were taken from the baseline study in Tromsø 4 in 1994–1995. Height and weight were measured in light clothing without shoes. Body mass index (BMI) was calculated as weight in kilograms divided by the square of height in metres. Blood pressure was assessed three times after 2 min seated rest with 1 min interval with an oscillometric digital automated device (Dinamap Vital Signs Monitor; Critikon Inc, Tampa, Florida, USA^21^). The mean systolic blood pressure of reading two and three was used. From non-fasting blood samples, total cholesterol level (mmol/L) and triglycerides (mmol/L) were analysed at the Department of Clinical Chemistry, University Hospital of North Norway. The participants completed two self-administered questionnaires, one before entering the study, and the other during the study. From these questionnaires, data on health status were derived (poor, not so good, good, very good), a history of heart attack, angina, stroke, asthma or diabetes. Participants also provided information on light physical activity (not sweating or out of breath) and hard physical activity (sweating and/or out of breath) during a typical week in the last year, and for each of the two variables participants were categorised in four groups based on hours per week in activity (none, less than one, one to two, three or more). Participants were asked if they used blood pressure-lowering drugs (currently, previously, but not now, never). Participants were dichotomised as current cigarette smokers or non-current cigarette smokers. Education was stratified into five groups: primary (7–10 years), technical school/middle school, high school diploma (3–4 years), college/university <4 years and college/university 4 years or more.

### Statistical methods

To allow investigation of variation in the association between grip strength and mortality by age, participants were assigned to one of the three age groups based on age at the time of grip strength assessment: 50–59, 60–69 and 70–80 years. Sex-specific quintiles for grip strength in each of these age groups were created. Age-specific and gender-specific grip strength z-scores were also created, which had a mean of 0 and SD of 1.

In a first set of analyses, Cox regression was used to assess the associations between fifths of grip strength and hazards of mortality, with the strongest fifth used as the reference category. In a second set of analyses, the associations were assessed using age-specific and gender-specific grip strength z-scores. In a third set of analyses, the associations were modelled using grip strength z-scores as natural cubic splines with three knots to allow for a more flexible analysis of grip strength on the continuous scale. This approach allowed us to investigate deviation from linearity, and test for evidence of thresholds. All three sets of analyses examined associations of grip strength with all-cause and cause-specific mortality rates. In all models, attained age was the time scale, and thereby models were adjusted for age. In addition, models were first adjusted for sex and body size (height and BMI) and then for all health-related and lifestyle-related factors. To investigate differences in associations by age group, an age group by grip strength interaction term was included in a subsequent stage of analyses. In the gender-adjusted model, gender was treated as a covariate. The proportional hazards assumption was checked using Schoenfeld residuals, and on the basis of visual inspection of the estimated hazard function. No severe violations of proportionality were observed. Stata SE V.14.0 was used for the analyses.

## Results

Mean value for the absolute grip strength for the total study population was 0.78 bar with SD 0.21, and 0.84 bar (SD 0.21) in men and 0.73 bar (SD 0.20) in women. By age group, the mean values in men were: 0.94 bar (SD 0.21) (50–59 years), 0.82 bar (SD 0.19) (60–69 years) and 0.70 bar (SD 0.17) (70–80 years). Corresponding values in women were: 0.78 bar (0.20), 0.70 bar (0.18) and 0.63 bar (0.15).

**Figure 1:**
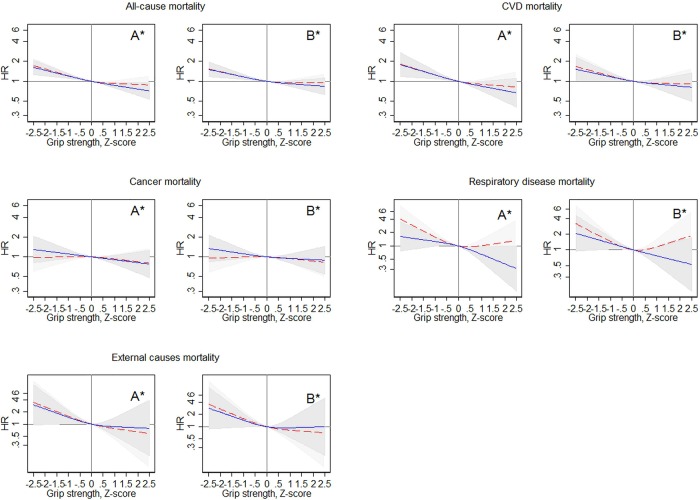
Grip z-scores and mortality HRs with 95% CIs (shaded areas) for men (blue solid lines) and women (red dashed lines). Estimated in Cox regression with grip z-scores modelled as natural cubic splines with three knots. Z-score 0 is reference. *A is minimally adjusted for age and body size (BMI and height); *B is fully adjusted for age and body size (BMI and height)+systolic blood pressure, total cholesterol, triglycerides, self-reported general health status, self-reported history of heart attack, stroke, angina, asthma and diabetes, self-reported blood pressure treatment, smoking, leisure-time physical activity and education. BMI, body mass index; CVD, cardiovascular disease.

Table 1 displays the baseline characteristics of the participants stratified by fifths of grip strength by gender. Those in the higher grip strength categories had higher BMI, were younger, reported better overall health, had less history of heart disease and asthma, smoked less, were more physically active and had higher education than those in the lower grip strength categories (all p<0.05). Overall, there were 2338 deaths among the 6850 participants during follow-up, of which 35% of deaths were caused by CVD (8% of which were stroke and 17% IHD), 35% by cancer, 9% by respiratory diseases, 3% by external causes (table 2) and 18% other causes. Regression models in tables 3 and 4 and in figure 1 were run on a slightly smaller sample with no missing data on covariates (N=6601, with 2225 deaths). Of these deaths, 776 were due to CVD (of which 183 were due to stroke and 366 were due to IHD), 774 were due to cancer, 200 were due to respiratory diseases, 68 were due to external causes and 407 were due to other causes. Minimally adjusted results on the maximum available samples produced very similar results (results not shown).

**Table 1:** Baseline characteristics of the Tromsø Study population, by grip strength fifths (gender and age specific)

	N	Total	1 (Lowest grip)	2	3	4	5 (Highest grip)	Trend*
Men								
Number of participants	2858	2858	593	562	627	514	562	
Number of deaths	1253	1253	317	258	266	214	198	
Mortality rate (95% CI)†	2858	38.6 (30.7 to 34.3)	43.6 (39.1 to 48.7)	34.4 (30.4 to 38.8)	31.1 (27.6 to 35.1)	30.1 (26.3 to 34.4)	24.4 (21.2 to 28.0)	<0.001‡
Age	2858	62.0 (6.4)	63.3 (6.4)	63.0 (6.0)	62.4 (6.3)	62.8 (6.5)	62.0 (6.6)	0.001
Grip strength (bar)	2858	0.84 (0.21)	0.58 (0.12)	0.74 (0.08)	0.85 (0.09)	0.95 (0.10)	1.12 (0.15)	<0.001
BMI (kg/m^2^)	2855	26.1 (3.4)	25.5 (3.7)	25.7 (3.4)	26.2 (3.2)	26.1 (3.1)	26.9 (3.3)	<0.001
Height (cm)	2855	174.8 (6.0)	172.4 (6.8)	174.0 (6.9)	174.7 (6.4)	176.0 (6.5)	177.2 (6.0)	<0.001
Systolic BP (mm Hg)	2857	146.7 (20.8)	146.0 (21.1)	145.2 (20.7)	147.5 (21.0)	146.1 (20.3)	148.7 (20.9)	0.019
Cholesterol (mmol/L)	2851	6.6 (1.2)	6.6 (1.2)	6.7 (1.2)	6.6 (1.2)	6.5 (1.1)	6.6 (1.2)	0.475
Triglycerides (mmol/L)	2847	1.8 (1.1)	1.7 (1.1)	1.8 (1.0)	1.8 (1.2)	1.7 (0.9)	1.9 (1.1)	0.158
Poor/not good health, %	2854	43	52	46	40	43	35	<0.001
History of heart attack, %	2848	10	10	11	10	13	6	0.006
History of angina, %	2847	12	16	13	11	12	9	0.017
History of stroke, %	2846	3	6	3	2	4	2	<0.001
History of asthma, %	2846	7	7	9	7	7	7	0.614
History of diabetes, %	2846	4	5	2	4	4	3	0.068
Blood pressure medication, %	2841	20	21	18	21	21	17	0.313
Smoke cigarettes, %	2857	31	35	36	33	29	24	<0.001
No light physical activity, %	2841	26	29	28	23	24	23	0.034
No hard physical activity, %	2827	73	80	74	68	74	71	<0.001
Low education, %	2847	48	57	50	49	44	38	<0.001
Women								
Number of participants	3992	3992	843	880	732	787	750	
Number of deaths	1085	1085	281	268	181	180	175	
Mortality rate (95% CI)†	3992	18.0 (16.9 to 19.1)	22.8 (20.3 to 25.6)	20.4 (18.1 to 23.0)	16.1 (13.9 to 18.6)	14.9 (12.9 to 17.3)	15.1 (13.0 to 17.5)	<0.001‡
Age	3992	60.8 (7.4)	61.2 (7.4)	61.1 (7.4)	60.6 (7.2)	60.5 (7.3)	60.2 (7.5)	0.001
Grip strength (bar)	3992	0.73 (0.20)	0.47 (0.11)	0.65 (0.06)	0.74 (0.06)	0.83 (0.07)	1.00 (0.12)	<0.001
BMI (kg/m^2^)	3984	26.1 (4.4)	26.1 (4.8)	25.7 (4.5)	26.0 (4.3)	26.3 (4.0)	26.4 (4.4)	0.011
Height (cm)	3986	161.6 (6.2)	160.5 (6.3)	161.1 (6.4)	161.5 (6.3)	161.9 (5.8)	163.1 (5.8)	<0.001
Systolic BP (mm Hg)	3991	144.8 (23.6)	142.8 (22.8)	144.7 (23.9)	145.5 (23.9)	145.0 (24.2)	146.3 (23.0)	0.006
Cholesterol (mmol/L)	3998	7.0 (1.3)	7.1 (1.3)	7.0 (1.3)	7.0 (1.2)	6.9 (1.2)	6.9 (1.3)	0.007
Triglycerides (mmol/L)	3986	1.6 (1.0)	1.6 (0.9)	1.6 (0.9)	1.6 (1.1)	1.6 (1.0)	1.6 (0.9)	0.692
Poor/not good health, %	3986	50	69	49	45	45	39	0.178
History of heart attack, %	3979	3	4	2	2	4	2	0.005
History of angina, %	3983	7	10	6	5	6	5	0.001
History of stroke, %	3976	2	4	2	1	1	0.5	<0.001
History of asthma, %	3975	9	11	10	7	7	7	0.008
History of diabetes, %	3976	3	3	4	2	2	3	0.294
Blood pressure medication, %	3975	17	19	17	16	17	17	0.609
Smoke cigarettes, %	3988	30	34	33	30	28	27	0.005
No light physical activity, %	3985	27	34	30	25	23	22	<0.001
No hard physical activity, %	3948	86	91	86	84	84	82	<0.001
Low education, %	3961	61	69	63	59	59	56	<0.001

Figures are means (SD), unless stated otherwise. For brevity, descriptive statistics for only the ‘highest risk’ category of each categorical covariate are presented. Categorisations used are as follows: self-reported health status (poor, not so good, good, very good); history of heart attack (yes, no); history of angina (yes, no); history of stroke (yes, no); history of asthma (yes, no); history of diabetes (yes, no); use of blood pressure-lowering drugs (currently, previously but not now, never); current cigarette smokers (yes, no); light physical activity such as not sweating or out of breath during a typical week in the last year (none, less than one, one to two, three or more); hard physical activity such as sweating and/or out of breath during a typical week in the last year (none, less than one, one to two, three or more); education (primary (7–10 years), technical school/middle school, high school diploma (3–4 years), college/university <4 years, college/university 4 years or more).

*p Values for trend based on linear regression for the continuous variables, and logistic regression for the dichotomous ones. Grip in fifths treated as a continuous variable in the regression models.

†Mortality rate per 1000 (95% CI).

‡The trend across mortality rates calculated using Poisson regression adjusted for age and sex.

BMI, body mass index; BP, blood pressure.

**Table 2:** Number of deaths and mortality rate (per 1000 person-years) by cause of death, gender and age group

	Men and women	Men				Women			
	50–80 years (N=6601)	50–80 years (N=2744)	50–59 years (N=987)	60–69 years (N=1259)	70–80 years (N=498)	50–80 years (N=3857)	50–59 years (N=1860)	60–69 years (N=1405)	70–80 years (N=592)
Number of deaths									
Total mortality	2225	1189	204	597	388	1036	218	456	362
Cardiovascular	776	423	71	201	151	353	44	148	161
Stroke	183	81	5	49	27	102	11	43	48
IHD	366	228	48	92	88	138	18	61	59
Cancer	774	412	86	219	107	362	125	168	69
Respiratory	200	115	15	55	45	85	17	39	29
External	68	39	–	–	–	29	–	–	–
Mortality rate									
Total mortality	23.3	31.9	13.5	35.6	73.0	17.7	7.3	21.8	48.5
Cardiovascular	8.1	11.4	4.7	12.0	28.4	6.0	1.5	7.1	21.6
Stroke	1.9	2.2	0.3	2.9	5.1	1.7	0.4	2.1	6.4
IHD	3.8	6.1	3.2	5.5	16.5	2.4	0.6	2.9	7.9
Cancer	8.1	11.1	5.7	13.1	20.1	6.2	4.2	8.0	9.2
Respiratory	2.1	3.1	1.0	3.3	8.5	1.5	0.6	1.9	3.9
External	0.7	1.0	–	–	–	0.5	–	–	–

Deaths due to external causes are only presented for 50–80 years and not age specific due to small numbers. Study sample has non-missing values for all included covariates (age, sex, BMI, systolic blood pressure, total cholesterol, triglycerides, self-reported general health status, self-reported history of heart attack, stroke, angina, asthma and diabetes, self-reported blood pressure treatment, smoking, leisure-time physical activity and education).

BMI, body mass index; IHD, ischaemic heart disease.

**Table 3:** HR (95% CI) for all-cause and cause-specific mortality per 1 SD reduction in grip strength

	Men and women	Men	Women	Gender interaction
Number of participants	6601	2744	3857	
*Cause of death ( number of deaths)*				
Model 1*				
Total mortality (2225)	1.17 (1.12 to 1.22)	1.18 (1.11 to 1.26)	1.16 (1.09 to 1.23)	0.91
Cardiovascular (776)	1.21 (1.12 to 1.30)	1.22 (1.10 to 1.36)	1.19 (1.07 to 1.33)	0.98
Stroke (183)	1.26 (1.08 to 1.47)	1.35 (1.06 to 1.71)	1.22 (0.99 to 1.49)	0.76
IHD (366)	1.18 (1.06 to 1.32)	1.17 (1.02 to 1.35)	1.20 (1.01 to 1.43)	0.59
Cancer (774)	1.07 (1.00 to 1.15)	1.11 (1.00 to 1.29)	1.04 (0.93 to 1.15)	0.46
Respiratory (200)	1.35 (1.16 to 1.57)	1.34 (1.10 to 1.63)	1.39 (1.11 to 1.74)	0.69
External causes (68)	1.44 (1.13 to 1.84)	1.37 (0.98 to 1.91)	1.51 (1.05 to 2.17)	0.79
Model 2†				
Total mortality (2225)	1.12 (1.07 to 1.17)	1.13 (1.06 to 1.20)	1.11 (1.04 to 1.19)	0.85
Cardiovascular (776)	1.14 (1.06 to 1.23)	1.14 (1.03 to 1.27)	1.15 (1.03 to 1.29)	0.68
Stroke (183)	1.19 (1.02 to 1.39)	1.20 (0.95 to 1.53)	1.18 (0.95 to 1.45)	0.93
IHD (366)	1.11 (1.00 to 1.24)	1.11 (0.96 to 1.28)	1.15 (0.96 to 1.37)	0.41
Cancer (774)	1.05 (0.97 to 1.13)	1.09 (0.98 to 1.21)	1.03 (0.92 to 1.15)	0.40
Respiratory (200)	1.23 (1.06 to 1.44)	1.32 (1.06 to 1.65)	1.22 (0.96 to 1.55)	0.48
External causes (68)	1.37 (1.06 to 1.76)	1.30 (0.92 to 1.83)	1.47 (0.99 to 2.17)	0.79

Age 50–80 years at baseline.

*Model 1. Adjusted for age, sex and body size (BMI and height).

†Model 2. Adjusted for age, sex and body size (BMI and height)+systolic blood pressure, total cholesterol, triglycerides, self-reported general health status, self-reported history of heart attack, stroke, angina, asthma and diabetes, self-reported blood pressure treatment, smoking, leisure-time physical activity and education.

BMI, body mass index; IHD, ischaemic heart disease.

**Table 4:** HR for all-cause and cause-specific mortality per 1 SD reduction in grip strength, by age at grip assessment

Cause of death (number of deaths in ages: 50–59, 60–69, 70–80 years)	Age 50–59	Age 60–69	Age 70–80	Age interaction
Model 1*				
Total mortality (422, 1053, 750)	1.21 (1.10 to 1.34)	1.18 (1.10 to 1.25)	1.13 (1.05 to 1.22)	0.69
Cardiovascular (115, 349, 312)	1.19 (0.98 to 1.45)	1.22 (1.09 to 1.36)	1.24 (1.10 to 1.39)	0.84
Cancer (211, 387, 176)	1.15 (1.00 to 1.32)	1.06 (0.96 to 1.18)	0.98 (0.84 to 1.16)	0.29
Model 2†				
Total mortality (422, 1053, 750)	1.14 (1.03 to 1.26)	1.13 (1.06 to 1.20)	1.07 (0.99 to 1.16)	0.61
Cardiovascular (115, 349, 312)	1.14 (0.94 to 1.39)	1.14 (1.02 to 1.28)	1.16 (1.02 to 1.31)	0.87
Cancer (211, 387, 176)	1.11 (0.96 to 1.27)	1.04 (0.93 to 1.15)	0.97 (0.82 to 1.14)	0.32

Number of participants: 2847 in ages 50–69 years, 2664 in ages 60–69 years and 1090 in ages 70–80 years.

*Model 1. Adjusted for age, sex and body size (BMI and height).

†Model 2. Adjusted for age, sex and body size (BMI and height)+systolic blood pressure, total cholesterol, triglycerides, self-reported general health status, self-reported history of heart attack, stroke, angina, asthma and diabetes, self-reported blood pressure treatment, smoking, leisure-time physical activity and education.

BMI, body mass index.

### All-cause mortality

Grip strength was inversely associated with all-cause mortality rates in men and women (table 3), with no grip strength by gender interaction found when grip strength was modelled linearly (p=0.91); 1 SD lower grip strength was associated with an HR of all-cause mortality of 1.17 (95% CI 1.12 to 1.22) in a model adjusted for age, gender and body size, which was only partially attenuated in the fully adjusted model (HR=1.12, 95% CI 1.07 to 1.17). The spline models suggested a linear association between grip and all-cause mortality rates in men, while in women, there was some evidence that only grip strength below the mean was associated with increased mortality rates (figure 1). Weaker grip strength was associated with increased hazards of mortality in all age groups (overall p<0.05; table 4), with no evidence of a grip strength by age interaction (p=0.69; table 4). Results were similar using grip in fifths rather than z-scores (see online supplementary appendix table A1).

10.1136/jech-2015-206776.supp1 Supplementary table A1 All-cause and cause specific mortality hazard ratios (HR) by grip strength quintiles and age groups. Estimated in Cox regression.

### Cardiovascular mortality

Weaker grip strength was associated with increased CVD mortality rates (per 1 SD lower grip strength HR=1.21, 95% CI 1.12 to 1.30) in a model adjusted for age, sex and body size, and this was not completely attenuated in the fully adjusted model (HR=1.14, 95% CI 1.06 to 1.22) (table 4). The association between grip strength and CVD mortality was similar in men and women (p=0.98). Furthermore, the association between grip strength and CVD mortality was similar across age groups (grip strength by age interaction, p=0.84) (table 4). Results were similar using grip in fifths rather than z-scores (see online supplementary appendix table A1). The spline models indicated linear associations between grip strength and CVD mortality (figure 1). Similar associations of grip strength with stroke and IHD mortality were found (table 3).

### Cancer mortality

Weaker grip strength was associated with increased cancer mortality in the minimally adjusted model (per 1 SD lower grip strength HR=1.07, 95% CI 1.00 to 1.16), but this was fully attenuated in the fully adjusted model (HR=1.05, 95% 0.97, 1.13). While there was some evidence in basic models that grip strength was associated with cancer mortality in men (HR=1.11, 95% CI 1.00 to 1.29) but not in women (HR=1.04, 95% CI 0.93 to 1.15), the grip strength by gender interaction term was not significant (p=0.46). However, after adjustment for health-related and lifestyle-related factors, the grip strength–cancer association was not significant in either sex (table 3, figure 1). Furthermore, there was evidence that the association between grip strength and cancer mortality was only significant in the youngest age group (ie, 50–59 years; HR=1.15, 95% CI 1.00 to 1.32), while in the older groups, the association did not reach statistical significance (table 4). However, the interaction between grip strength and age group was non-significant (p=0.29; table 4). Results were similar using grip in fifths rather than z-scores (see online supplementary appendix table A1).

### Respiratory and external causes of mortality

Weaker grip strength was significantly associated with increased respiratory disease mortality in the minimally adjusted model (per 1 SD lower grip strength HR=1.35, 95% CI 1.16 to 1.57) (table 3), and the HR did not differ between men and women (p=0.69). The association was robust to adjustment for a range of health-related factors (HR=1.23, 95% CI 1.06 to 1.44). For external causes of death, a 1 SD lower grip strength was associated with HR=1.44 (95% CI 1.13 to 1.84) in the minimally adjusted model, and there was no significant grip strength by gender interaction (p=0.79). The association was robust to further adjustment (HR=1.37, 95% CI 1.06 to 1.76). Numbers of deaths due to respiratory diseases and external factors were too small for age-specific analyses of these causes of death. See figure 1 for a graphical illustration of the associations of grip strength with deaths from respiratory diseases and external causes.

Results for all-cause mortality, CVD mortality and cancer mortality using grip in fifths are reported in online supplementary appendix table A1.

There were 162 participants reporting a history of stroke (2.4%), and as a sensitivity analysis, we performed an additional set of analyses in which these participants were excluded. This sensitivity analysis provided results similar to those described above.

## Discussion

In this large population-based study of home-dwelling Norwegians aged 50–80 years followed over 17 years including 2338 deaths, we found weaker grip strength to be associated with significantly higher rates of all-cause mortality in a stepwise fashion. This association was similar across age groups and in men and women. These associations were not fully explained by adjustment for a range of lifestyle-related and health-related factors. Results for deaths due to CVD, respiratory disease and external causes resembled those for all-cause mortality, while cancer mortality was only weakly associated with grip strength in men, and this association was not significant in fully adjusted analyses.

The finding of an association between weaker grip strength and higher rates of all-cause mortality is in line with findings from previous reports.^9–13^ Our finding of similar associations between grip strength and all-cause mortality across age groups is in line with the few previous reports which have been able to stratify analyses by age.^14–16^ ^19^ ^22^ ^23^ Our finding of an equally strong inverse association between grip strength and all-cause mortality in men and women is also consistent with findings in other studies.^14^ ^17^

Analyses of grip strength and cause-specific mortality are less numerous.^14–19^ Consistent with our findings, those six studies which have explored cause-specific mortality previously have found stronger grip to be associated with lower CVD mortality rates,^14–19^ and those four studies which included men and women found the association to be similar in both genders.^14–17^ The two studies that had investigated stroke mortality found similarly to us that this association was similar to the grip strength—CVD association. The consistency across studies of the inverse association between grip strength and CVD mortality suggests potential mechanism acting on the cardiovascular pathway. Increased grip strength has been found to be associated with a more favourable cardiovascular risk factor profile,^24^ but the strong association found in our study was only weakly attenuated after adjustment for a wide range of CVD risk factors. Another important finding in support of the cardiovascular pathway is that grip strength has been found to be an equally important prognostic factor as systolic blood pressure regarding CVD mortality risk.^14^

In our study, the association between grip strength and cancer mortality was weaker than for CVD, and restricted to men. Results for grip strength and cancer mortality are not consistent in the literature. In line with our findings, Sasaki *et al*^15^ and Gale *et al*^17^ found stronger grip to be associated with lower cancer mortality in men only. Another study of US men also found stronger grip to be associated with lower cancer mortality risk.^18^ The other two studies reported no significant association,^16^ ^19^ and Leong *et al*^14^ reported the unexpected finding of a protective effect of low grip strength on cancer mortality. Potential explanations for the inconsistent results regarding cancer mortality might be due to power issues and differences in study designs. Further studies should investigate the association by cancer subtype.

We had sufficient power to study the association between grip strength and mortality from respiratory diseases, and our results suggest that weak muscle strength is associated with deaths from respiratory diseases in men and women. To our knowledge, only two other studies have investigated this, and their results are in line with ours.^16^ ^17^ In contrast with these findings, no association between grip strength and hospital admission with respiratory illness was reported in a recent study.^14^ However, this could be due to differences in study outcome, that is, hospital admission versus mortality. Another study from Japan reported an association between grip strength and death due to pneumonia, one important cause of respiratory death.^15^

Our finding of an association between weak grip strength and increased rates of death due to external causes is novel, and we are not aware of other studies investigating this. Suicide, a subcategory of external causes, was studied in male adolescents in a Swedish study, and here those in the lowest tenth of grip strength were found to have higher rates of mortality due to suicide.^19^ It was suggested that physically weaker men might be more psychologically vulnerable, but the mechanisms are not fully understood. In our study, suicide constituted only 8% of the deaths due to external causes, and results were similar when deaths due to external causes were examined having excluded those due to suicide (results not shown). The absence of an association between grip strength and the risk of injury from falls (another subcategory of deaths due to external causes in our study) was surprising to the authors in another study.^14^ A large share of deaths due to external causes in the age group we have studied are falls related, about one in three for ages above 75 years and one in four in the age group 70–74 years.^25^ Thus, our findings contrast those of the other study,^14^ and this divergence could be due to the relatively young age in the other study (39–74 years at the end of follow-up) compared with our participants (52–96 years at the end of follow-up). It could also be due to differences in study outcome, that is, hospital admission versus mortality.

Most studies on grip strength and cause-specific mortality have been performed among older populations, but associations have also been shown for grip strength assessed at younger ages^19^ ^26^ and middle age.^9^ ^13^ ^16^ Our results of similar associations between grip strength and all-cause mortality and CVD mortality in midlife and old age are in line with results from Japan^16^ and the USA.^18^ Studies restricted to all-cause mortality also show similar association with mortality, whether grip was measured in midlife or old age.^9^

Our finding that grip strength is linked to deaths from diseases that are strongly associated with ageing in our study, such as CVD, stroke and respiratory diseases, is consistent with the explanation that grip strength is a marker of underlying ageing and disease processes. The strong association with deaths due to external causes, such as falls, is also likely to be connected to the ageing process, for example, decreasing reaction time and reduced postural control. Thus, these findings suggest that weak grip strength is a marker for advanced biologic age and poor overall health. However, our results were robust and not fully explained by adjustment for a range of health-related factors, which suggests that poor overall health does not fully explain our findings. Furthermore, our consistent findings across all age groups, including the youngest age group, suggest that clinically manifest conditions are unlikely to provide the whole explanation. It suggests that weak grip strength may indicate underlying ageing and disease processes even prior to their clinical manifestation and thus may be a useful early sentinel of later life risk.

The main strengths of the present population-based study are the long follow-up time and the large sample size and high number of deaths, with sufficient statistical power to perform cause-specific analyses stratified by gender and age groups. The Cause of Death Registry has a near-complete coverage and the data quality is good.^27^ Another strength is our novel finding of an association between grip strength and mortality from external causes, which we were able to show was not driven by the association between grip strength and suicide, which has previously been reported.^19^ A limitation of our study is also related to study size, as it is underpowered to break down the rather broad group of ‘external causes’ into finer subgroups. Another limitation is our use of a single measurement of grip strength, whereby we were unable to study change in grip strength and its association with mortality, which has been reported previously.^28^ As in most observational studies, there is also the possibility of unmeasured confounding, which may account for the relationship between grip strength and mortality. In our study, we report age-specific and gender-specific grip strength z-scores; therefore, the age and gender by grip interactions are based on mortality HRs associated with a 1 unit z-score change within each combination of age group and sex. If the grip strength distribution varied markedly between groups, for example, between age groups, a 1 z-score unit change would mean a quite different absolute change in grip strength for young and old, which could affect interpretation of results. While the SD was somewhat smaller in the older age group, it was similar in both genders, and when an additional set of analyses were run using absolute values of grip measured in bar, these gave results similar to the analyses using z-scores (results not shown).

In conclusion, weaker grip strength was significantly associated with increased rates of all-cause mortality and deaths due to a range of causes, including CVD, stroke, IHD and respiratory diseases. A novel finding was the association between weaker grip strength and deaths due to external causes. These associations were similar in both genders and across age groups, which supports the hypothesis that grip strength might be a useful biomarker of ageing over the lifespan.

**What is already known on this subject**

- Most studies of grip strength in middle and old age have found a protective effect of high grip strength on all-cause mortality.
- Studies that have examined cause-specific mortality are not as numerous, and there is a lack of studies that have investigated age differences in these associations.
- Often studies have been powered only to investigate broad groups of causes of death such as cardiovascular disease and cancer, and have not been able to look at additional causes such as ischaemic heart disease, respiratory diseases and external causes.

**What this study adds**

- In line with previous studies, weaker grip strength was associated with increased rates of all-cause mortality and mortality due to cardiovascular disease (CVD) and respiratory diseases.
- One study has reported weaker grip strength in adolescence to be associated with increased rates of death from suicide among men, but our study is the first to report an association of grip strength with mortality due to a broader range of external causes in both sexes across different age groups.
- A much weaker association was observed for cancer-related deaths than for all-cause and CVD mortality.
- These associations were similar in both genders and across age groups, which supports the hypothesis that grip strength might be a biomarker of ageing over the lifespan.

## References

[1] Bohannon RW Muscle strength: clinical and prognostic value of hand-grip dynamometry. Curr Opin Clin Nutr Metab Care 2015;18:465–70. 10.1097/MCO.0000000000000202 26147527

[2] Roberts HC, Denison HJ, Martin HJ, et al A review of the measurement of grip strength in clinical and epidemiological studies: towards a standardised approach. Age Ageing 2011;40:423–9. 10.1093/ageing/afr051 21624928

[3] Rantanen T, Masaki K, Foley D, et al Grip strength changes over 27 yr in Japanese-American men. J Appl Physiol (1985) 1998;85:2047–53. 9843525 10.1152/jappl.1998.85.6.2047

[4] Cooper R, Hardy R, Aihie Sayer A, et al Age and gender differences in physical capability levels from mid-life onwards: the harmonisation and meta-analysis of data from eight UK cohort studies. PLoS ONE 2011;6:e27899 10.1371/journal.pone.0027899 22114723 PMC3218057

[5] Metter EJ, Conwit R, Tobin J, et al Age-associated loss of power and strength in the upper extremities in women and men. J Gerontol A Biol Sci Med Sci 1997;52:B267–76. 10.1093/gerona/52A.5.B267 9310077

[6] Dodds RM, Syddall HE, Cooper R, et al Grip strength across the life course: normative data from twelve British studies. PLoS ONE 2014;9:e113637 10.1371/journal.pone.0113637 25474696 PMC4256164

[7] Sayer AA, Kirkwood TB Grip strength and mortality: a biomarker of ageing? Lancet 2015;386:226–7. 10.1016/S0140-6736(14)62349-7 25982159

[8] Cooper R, Kuh D, Cooper C, et al Objective measures of physical capability and subsequent health: a systematic review. Age Ageing 2011;40:14–23. 10.1093/ageing/afq117 20843964 PMC3000177

[9] Cooper R, Kuh D, Hardy R Objectively measured physical capability levels and mortality: systematic review and meta-analysis. BMJ 2010;341:c4467 10.1136/bmj.c4467 20829298 PMC2938886

[10] Koopman JJ, van Bodegom D, van Heemst D, et al Handgrip strength, ageing and mortality in rural Africa. Age Ageing 2015;44:465–70. 10.1093/ageing/afu165 25331975 PMC4411221

[11] Matos CM, Silva LF, Santana LD, et al Handgrip strength at baseline and mortality risk in a cohort of women and men on hemodialysis: a 4-year study. J Ren Nutr 2014;24:157–62. 10.1053/j.jrn.2013.12.005 24598143

[12] Martín-Ponce E, Hernández-Betancor I, González-Reimers E, et al Prognostic value of physical function tests: hand grip strength and six-minute walking test in elderly hospitalized patients. Sci Rep 2014;4:7530 10.1038/srep07530 25531922 PMC4273599

[13] Cooper R, Strand BH, Hardy R, et al Physical capability in mid-life and survival over 13 years of follow-up: British birth cohort study. BMJ 2014;348:g2219 10.1136/bmj.g2219 24787359 PMC4004787

[14] Leong DP, Teo KK, Rangarajan S, et al Prognostic value of grip strength: findings from the Prospective Urban Rural Epidemiology (PURE) study. Lancet 2015;386:266–73. 10.1016/S0140-6736(14)62000-6 25982160

[15] Sasaki H, Kasagi F, Yamada M, et al Grip strength predicts cause-specific mortality in middle-aged and elderly persons. Am J Med 2007;120:337–42. 10.1016/j.amjmed.2006.04.018 17398228

[16] Kishimoto H, Hata J, Ninomiya T, et al Midlife and late-life handgrip strength and risk of cause-specific death in a general Japanese population: the Hisayama Study. J Epidemiol Community Health 2014;68:663–8. 10.1136/jech-2013-203611 24622276

[17] Gale CR, Martyn CN, Cooper C, et al Grip strength, body composition, and mortality. Int J Epidemiol 2007;36:228–35. 10.1093/ije/dyl224 17056604

[18] Ruiz JR, Sui X, Lobelo F, et al Association between muscular strength and mortality in men: prospective cohort study. BMJ 2008;337:a439 10.1136/bmj.a439 18595904 PMC2453303

[19] Ortega FB, Silventoinen K, Tynelius P, et al Muscular strength in Male adolescents and premature death: cohort study of one million participants. BMJ 2012;345:e7279 10.1136/bmj.e7279 23169869 PMC3502746

[20] Jacobsen BK, Eggen AE, Mathiesen EB, et al Cohort profile: the Tromsø Study. Int J Epidemiol 2012;41:961–7. 10.1093/ije/dyr049 21422063 PMC3429870

[21] Hopstock LA, Bonaa KH, Eggen AE, et al Longitudinal and secular trends in blood pressure among women and men in birth cohorts born between 1905 and 1977: the Tromsø Study 1979 to 2008. Hypertension 2015;66:496–501. 10.1161/HYPERTENSIONAHA.115.05925 26195482

[22] Rantanen T, Harris T, Leveille SG, et al Muscle strength and body mass index as long-term predictors of mortality in initially healthy men. J Gerontol A Biol Sci Med Sci 2000;55:M168–73. 10.1093/gerona/55.3.M168 10795731

[23] Metter EJ, Talbot LA, Schrager M, et al Skeletal muscle strength as a predictor of all-cause mortality in healthy men. J Gerontol A Biol Sci Med Sci 2002;57:B359–65. 10.1093/gerona/57.10.B359 12242311

[24] Lawman HG, Troiano RP, Perna FM, et al Associations of Relative Handgrip Strength and Cardiovascular Disease Biomarkers in U.S. Adults, 2011–2012. Am J Prev Med 2015;50:677 83. 10.1016/j.amepre.2015.10.022 26689977 PMC7337414

[25] Statistics Norway Statistics Bank. Oslo, Norway: Statistics Norway, 2012.

[26] Silventoinen K, Magnusson PK, Tynelius P, et al Association of body size and muscle strength with incidence of coronary heart disease and cerebrovascular diseases: a population-based cohort study of one million Swedish men. Int J Epidemiol 2009;38:110–18. 10.1093/ije/dyn231 19033357

[27] Pedersen AG, Ellingsen CL Data quality in the causes of death registry. Tidsskr Nor Laegeforen 2015;135:768–70. 10.4045/tidsskr.14.1065 25947599

[28] Xue QL, Beamer BA, Chaves PH, et al Heterogeneity in rate of decline in grip, hip, and knee strength and the risk of all-cause mortality: the Women's Health and Aging Study II. J Am Geriatr Soc 2010;58:2076–84. 10.1111/j.1532-5415.2010.03154.x 21054287 PMC3058914

