# Supplementary material for: The association of grip strength from midlife onwards with all-cause and cause-specific mortality over 17 years of follow-up in the Tromsø Study
Source: J Epidemiol Community Health. 2016 May 26;70(12):1214–21. doi: 10.1136/jech-2015-206776 (PMC5136688; doi:10.1136/jech-2015-206776)
Supplement: Supplementary table A1 — All-cause and cause specific mortality hazard ratios (HR) by grip strength quintiles and age groups. Estimated in Cox regression. [file jech-2015-206776supp_table.pdf]

APPENDIX

Table A1. All-cause and cause specific mortality hazard ratios (HR) by grip strength quintiles and age groups. Estimated in Cox regression.

	50-59 years*	60-69 years*	70-80 years*	50-80 years*	50-80 years**
Fifths of grip	**All-cause mortality**				
Low	1.64 (1.21, 2.24)	1.52 (1.25, 1.83)	1.47 (1.18, 1.85)	1.52 (1.33, 1.74)	1.33 (1.16, 1.52)
2	1.21 (0.88, 1.67)	1.29 (1.06, 1.58)	1.32 (1.06, 1.65)	1.27 (1.11, 1.46)	1.13 (0.98, 1.30)
3	1.11 (0.80, 1.54)	1.15 (0.94, 1.41)	1.15 (0.91, 1.45)	1.13 (0.99, 1.30)	1.04 (0.98, 1.30)
4	1.06 (0.75, 1.49)	1.00 (0.81, 1.23)	1.19 (0.95, 1.50)	1.08 (0.94, 1.25)	1.00 (0.87, 1.16)
High	1	1	1	1	1
Overall p-value: 0.005		*<0.001*	*0.012*	*<0.001*	*<0.001*
Age by grip interaction: P=0.898					
	**CVD mortality**				
Low	1.37 (0.76, 2.48)	1.88 (1.33, 2.65)	1.80 (1.25, 2.58)	1.66 (1.32, 2.10)	1.39 (1.09, 1.77)
2	1.22 (0.67, 2.20)	1.59 (1.11, 2.27)	1.63 (1.15, 2.33)	1.52 (1.20, 1.92)	1.27 (1.00, 1.61)
3	1.20 (0.66, 2.16)	1.47 (1.03, 2.10)	1.31 (0.89, 1.92)	1.32 (1.04, 1.68)	1.18 (0.93, 1.51)
4	0.75 (0.37, 1.52)	1.20 (0.83, 1.75)	1.43 (0.99, 2.07)	1.18 (0.92, 1.52)	1.04 (0.81, 1.34)
High	1	1	1	1	1
Overall p-value: 0.444		*0.003*	*0.019*	*<0.001*	*0.035*
Age by grip interaction: P=0.827					
	**Cancer mortality**				
Low	1.78 (1.12, 2.81)	1.11 (0.81, 1.53)	1.14 (0.73, 1.78)	1.22 (0.97, 1.53)	1.15 (0.91, 1.45)
2	1.47 (0.92, 2.33)	1.27 (0.93, 1.75)	0.99 (0.64, 1.53)	1.19 (0.95, 1.49)	1.13 (0.90, 1.42)
3	1.28 (0.79, 2.07)	1.08 (0.79, 1.49)	0.70 (0.43, 1.14)	1.03 (0.81, 1.29)	0.98 (0.77, 1.23)
4	1.49 (0.93, 1.99)	1.00 (0.72, 1.38)	1.02 (0.66, 1.58)	1.12 (0.89, 1.41)	1.10 (0.87, 1.38)
High	1	1	1	1	1
Overall p-value: 0.156		*0.510*	*0.413*	*0.332*	*0.539*
Age by grip interaction=0.438					

* Adjusted by age, sex, and body size (BMI and height).
** Adjusted by age, sex, and body size (BMI and height) + systolic blood pressure, total cholesterol, triglycerides, self-reported general health status, self-reported history of heart attack, stroke, angina, asthma and diabetes, self-reported blood pressure treatment, smoking, leisure time physical activity, education.
